# Improving the Culture of Human Skin Explants for Use in Preclinical Testing of Wound Healing Treatments

**DOI:** 10.3390/pharmaceutics17121611

**Published:** 2025-12-15

**Authors:** Xiao Guo, Martina Hüging, Ursula Mirastschijski, Ulrike Blume-Peytavi, Annika Vogt, Christoph Schaudinn, Fiorenza Rancan

**Affiliations:** 1 Clinical Research Center for Hair and Skin Science, Department of Dermatology and Allergy, Charité-Universitaetsmedizin Berlin, 10117 Berlin, Germany; xiao.guo@charite.de (X.G.); ulrike.blume-peytavi@charite.de (U.B.-P.); annika.vogt@charite.de (A.V.); 2 Department of Pediatric Surgery including Pediatric Urology, Charité-Universitaetsmedizin Berlin, 13353 Berlin, Germany; martina.hueging@charite.de; 3 Department of Biology and Chemistry, University of Bremen, 28359 Bremen, Germany; prof.mira@mira-beau.de; 4 Advanced Light and Electron Microscopy, Center for Biological Threats and Special Pathogens 4, Robert Koch Institute, 13353 Berlin, Germany; schaudinnc@rki.de

**Keywords:** ex vivo human skin model, animal-free culture media, wound healing, fetal calf serum (FCS), normal human serum (NHS), oxygen carrier (OC), angiogenesis, extracellular matrix remodeling

## Abstract

**Background:** Cultured human skin explants provide preclinical models to investigate drug delivery and the efficacy of topical treatments for wound healing. However, different culture conditions may affect cell viability, proliferation, and even wound healing. Since animal-derived supplements can influence the investigation of human physiological responses, this study evaluated the effects of non-animal supplements on the ex vivo wound healing process to improve the use of this model for preclinical drug efficacy tests. **Methods:** In in vitro scratch assays using HaCaT cells and fibroblasts, for media supplemented with normal human serum (NHS), oxygen carriers (OCs) had a positive impact on cell migration, supporting the further evaluation in ex vivo skin culture models. Human skin explants with standardized superficial wounds were cultured in four supplemented media: (i) Dulbecco’s Modified Eagle Medium High Glucose (DMEM) with fetal calf serum (FCS), (ii) DMEM with NHS and OC, (iii) CnT-Prime^TM^ with NHS and OC, and (iv) EpiLife™ with NHS and an OC. **Results:** During the 12-day culture, we observed re-epithelialization in all groups with the exception of EpiLife + NHS + OC (with no Ca^++^ supplement). For these samples, starting from day 6, we noticed a loosening of the dermal–epidermal junction and disruption of the upper epidermal layer. Furthermore, an immunohistochemical analysis of extracellular matrix components and remodeling factors, including type I and III collagen, transforming growth factor-β2, and matrix metalloproteinase-9, provided insights into tissue repair dynamics. **Conclusions:** NHS plus OC is comparable to FCS supplementation and represents a more physiological and ethical alternative.

## 1. Background

Chronic wounds represent a considerable global health challenge, characterized by prolonged healing, high morbidity, and substantial healthcare costs [1]. The development of novel wound therapies, including topical drug delivery strategies, requires physiologically relevant preclinical models that accurately replicate the structure and function of human skin. However, many conventional in vitro and animal models fall short in reproducing key aspects of chronic wound dynamics.

To overcome these limitations, ex vivo human skin explant models have emerged as valuable platforms for wound healing research, offering several advantages over conventional in vitro and animal models. Unlike in vitro systems, which often rely on two-dimensional (2D) single-cell line or co-culture techniques, ex vivo models preserve many in vivo skin features and the native epidermal–dermal architecture, allowing for the study of complex, human-specific physiological responses [2]. Compared to animal models, ex vivo human skin better replicates the structural and cellular complexity of human tissue, avoiding interspecies variability and ethical concerns [3]. Ex vivo human skin explants are particularly valuable for evaluating the effects of therapeutics on skin-specific processes, such as barrier function, cellular interactions, and ECM remodeling. Additionally, while reconstructed skin models and organotypic cultures represent a significant step forward, their simplified microstructure, in particular the absence of many skin cell types, limits their applicability in replicating native skin processes [4]. In contrast, ex vivo skin explants, derived from donated human tissues, retain all major skin components, including keratinocytes, fibroblasts, melanocytes, Langerhans cells, mast cells, and the ECM constituent proteins such as elastin and collagen as well as enzymes like matrix metalloproteases (MMPs). These models provide a more accurate representation of wound healing dynamics, with evidence suggesting that they remain viable for at least 12 days under appropriate culture conditions [5]. The structural integrity and physiological relevance of ex vivo human skin models make them important tools for preclinical research in areas ranging from wound healing to radiation-induced damage, sensitization, inflammation, and the evaluation of topically applied drug delivery systems and dermatological interventions [6,7,8,9]. Specifically, because ex vivo skin explants lack the immune and hormonal signaling from the blood stream, they resemble the typical features of chronic wounds caused by a low or defective blood supply, e.g., diabetic and venous chronic ulcers.

The applicability of these models is often constrained by the challenge of maintaining long-term tissue viability. The choice of the culture medium, supplementation, and environmental factors, such as oxygenation, plays a primary role in sustaining tissue integrity and cellular functionality over extended periods. However, even after extensive research, the selection of an optimal culture medium remains complex and is often guided by empirical approaches rather than experimentally defined criteria [10]. Optimizing these culture conditions is therefore indispensable for improving the physiological relevance and reproducibility of results, ultimately enhancing the translational potential of ex vivo models for studying human wound healing responses and establishing reliable platforms for preclinical testing.

Nutrient availability is crucial in determining wound healing outcomes. Therefore, we selected three different basic media that are slightly different in terms of essential nutrients. Dulbecco’s Modified Eagle Medium High Glucose (DMEM) was chosen as the standard basic medium widely used for cell cultures. It contains a high concentration of glucose; amino acids; L-glutamine; vitamins; and inorganic salts like calcium chloride (1.8 mM), potassium chloride, magnesium sulfate, sodium chloride, and sodium phosphate. The CnT-Prime Airlift medium (CnT medium) contains a tailored mix of growth factors (of non-animal origin), lipids, amino acids, vitamins, and minerals. The concentration of calcium is low (0.07 mM) to optimize the long-term proliferation of epithelial progenitor cells. The EpiLife medium is a basal medium composed of essential and non-essential amino acids, vitamins, other organic compounds, trace minerals, and inorganic salts, but it does not contain calcium, which must be supplemented.

Traditionally, fetal calf serum (FCS) has been a foundation of cell cultures due to its composition, which is rich in growth factors, hormones, lipids, and other proteins that broadly support cellular proliferation, migration, and differentiation. However, FCS presents limitations, such as batch-to-batch variability and an undefined composition, which can compromise experimental reproducibility [11]. Furthermore, its animal origin raises ethical concerns and diminishes its physiological relevance in studies involving human ex vivo skin explants. In contrast, normal human serum (NHS) offers a more biologically relevant alternative for human-derived systems. NHS contains human-specific growth factors, albumin, and cytokines at physiological concentrations, closely mimicking the environment of human tissues [12]. While NHS also exhibits variability between batches, it supports more ethical research practices by reducing the reliance on animal-derived components, adhering to the principles of Replacement, Reduction, and Refinement (3Rs). Integrating NHS into culture protocols for ex vivo wound models supports a more physiologically relevant environment, improving the reliability and applicability of findings for translational research [13].

Another important aspect is oxygen availability. This is crucial for mitigating hypoxia-induced stress, sustaining cellular metabolism, and promoting keratinocyte migration and fibroblast-driven ECM remodeling. The incorporation of oxygen carriers (OCs) into culture media represents a novel strategy to enhance oxygen diffusion in 2D cell cultures and could potentially improve cell survival in 3D organoids or scaffolds [14]. For this reason, in this work we investigated the effect of HEMOXCell^®^, (Hemarina, Morlaix, France), an animal-free OC, in vitro and ex vivo culture experiments.

In this study, we conducted a comprehensive assessment of wound healing markers in ex vivo human skin explants cultured in three distinct medium formulations supplemented with NHS and OCs and compared them with skin cultured in conventional FCS-supplemented DMEM. Through this comparison, we sought to improve the physiological relevance of this model, establishing a robust platform for the preclinical screening of wound healing treatments. To our knowledge, this is the first study to comprehensively evaluate the dynamic interplay between re-epithelialization, vascularization, and ECM remodeling in an ex vivo human wound model under different culture conditions.

## 2. Materials and Methods

### 2.1. Primary Skin Fibroblast Isolation and Culture

Fibroblasts were isolated from healthy breast skin tissue obtained from donors who underwent cosmetic surgery, with informed written consent provided. The experiment adhered to the guidelines of the Declaration of Helsinki and was approved by the Ethics Committee of the Charité-Universitätsmedizin Berlin (approval EA1/382/20, 23 March 2021). Skin tissue samples were cut into 3 × 3 mm pieces and digested with 2.4 U/mL dispase at 4 °C overnight. Following digestion, the dermis and epidermis were separated. Dermal tissue pieces were further digested with 3 mg/mL collagenase (Biochrom, Berlin, Germany), 1.5 mg/mL hyaluronidase (Sigma-Aldrich, Hamburg, Germany), and 10 μg/mL DNAse (Roche Diagnostics, Penzberg, Germany) at 37 °C for 2 h using a Thermo Mixer set at 700 rpm. After digestion, the dermal tissue was thoroughly mixed and passed through a 70 µm cell strainer (Falcon™, Durham, NC, USA). Cells were centrifuged at 300× *g* for 15 min, yielding approximately 2 × 10^6^ cells, which were resuspended in DMEM (Lonza™, Verviers, Belgium) supplemented with 10% fetal calf serum (FCS; PAA, Heidelberg, Germany), 4.5 g/L glucose, 2 mM UltraGlutamine I, and 100 µg/mL streptomycin (Sigma-Aldrich, Hamburg, Germany). Subcultures were performed when cells reached approximately 80% confluence. In vitro wound scratch assays were conducted using fibroblasts from passages 4 to 7.

### 2.2. In Vitro Wound Scratch Assay

HaCaT keratinocytes (obtained from Deutsches Krebsforschungszentrum, Heidelberg, Germany) and primary skin fibroblasts were seeded separately in µ-Dish 35 mm cell culture dishes (Ibidi GmbH, Munich, Germany) at a concentration of 3 × 10^5^ cells/mL, using an Ibidi culture insert (Ibidi GmbH, Munich, Germany) to create two compartments. Following the manufacturer’s instructions, 70 μL of the cell suspension was added to each compartment. Cells were incubated at 37 °C in a humidified atmosphere with 5% CO_2_ for 24 h to allow adhesion and confluence. After incubation, the culture insert was gently removed to create the in vitro wound model. The cells were washed with phosphate-buffered saline (PBS, Thermo Fisher Scientific, Waltham, MA, USA) to remove debris, and fresh culture media with different supplements were added to each well as follows: (i) DMEM with 1% FCS, (ii) DMEM with 10% FCS, (iii) DMEM with 10% FCS and 0.025 mg/mL of an oxygen carrier (OC, HEMOXCell, Hemarina, France), (iv) DMEM with 10% normal human serum (NHS, Capricorn Scientific GmbH, Ebsdorfergrund, Germany), (v) DMEM with 10% NHS and 0.025 mg/mL OC, (vi) CnT medium (CnT-Prime Airlift medium, CELLnTEC Advanced Cell Systems AG, Bern, Switzerland) with 10% NHS, and (vii) CnT medium with 10% NHS and 0.025 mg/mL OC. HaCaT cells were used due to their donor independence, high reproducibility, and robust tolerance to changes in serum composition, making them well-suited for comparative migration assays. Note: Fetal calf serum (FCS), also commonly referred to as fetal bovine serum (FBS), is a widely used supplement in cell culture studies, with regional preferences influencing terminology [12]. In this study, we consistently use “FCS” as the abbreviation to refer to fetal calf serum. Images of cells that migrated into the wound area were captured at 0, 4, 8, 24, and 48 h using a confocal laser microscope (LSM 700, Carl Zeiss Ltd., Jena, Germany). All in vitro scratch assays were performed in triplicate. Wound closure was quantified using ImageJ software, version 1.47 (National Institutes of Health, Bethesda, MD, USA). The percentage of wound closure was calculated using the following formula: Wound closure (%) = [(Initial wound area−Wound area at time n)/Initial wound area] × 100%. The wound healing speed was calculated using the following formula: Healing speed (μm^2^/h) = (Initial wound area−Wound area at time n)/Time span.

### 2.3. Ex Vivo Skin and Superficial Wound Model

Human skin was obtained from three female donors (mean age: 43) undergoing abdominoplasty, with full informed written consent. Ethical approval for the study was granted by the Ethics Committee of the Charité-Universitätsmedizin Berlin (approval EA1/382/20, 23 March 2021). Skin samples were processed within 2–4 h post-surgery and were selected based on criteria ensuring intact skin without stretch marks, scars, or signs of infection. Skin samples were cut into 1.5 × 1.5 cm pieces, retaining a 1–2 mm layer of subcutaneous adipose tissue, and blocked onto Parafilm-covered Styrofoam blocks using syringe needles. Prior to wound creation, the skin was cleaned topically with sterile 0.9% saline solution. A superficial wound was created by removing the epidermis using a ball-shaped milling cutter (No. 28725, Proxxon, Föhren, Germany) mounted on a micro-motor handpiece (Marathon N7, TPC Advanced Technology, Inc., City of Industry, CA, USA) operating at 16,000 rpm. This process produced a standardized wound with a diameter of 3 mm.

### 2.4. Skin Culture Procedure

**Figure 1:**
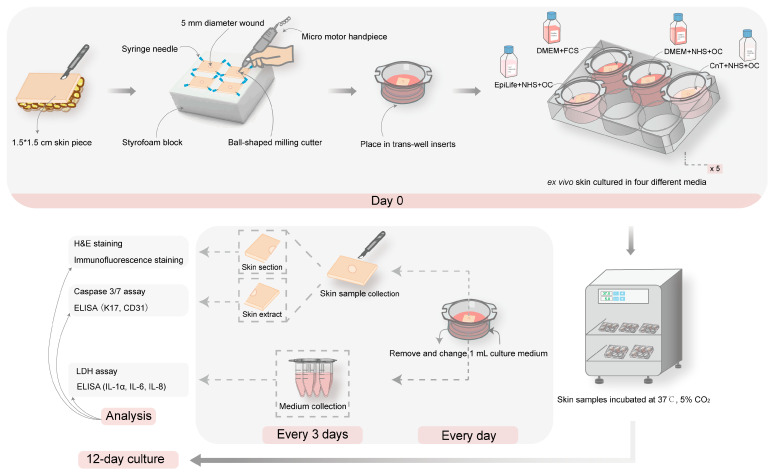
A schematic representation of the standardized ex vivo wound model and of the experimental design. Skin samples were cut into 1.5 × 1.5 cm pieces, and superficial wounds with a diameter of 3 mm were created. The wounded skin samples were transferred into inserts and cultured in four different media with different supplementation: (i) DMEM with FCS, (ii) DMEM with NHS and OC, (iii) CnT medium with NHS and OC, and (iv) EpiLife™ medium with NHS and OC. A total of five plates containing skin samples were cultured at 37 °C, 5% CO_2_ for 1, 3, 6, 9, and 12 days. At 3-day intervals, one skin sample and 1 mL of culture medium per group were collected. One half of the skin samples was used for histology (H&E staining), immunofluorescence staining (Keratin 17 (K17) and CD31), and immunohistochemical (IHC) staining (type I and type III collagen, TGFβ2, and MMP-9). The other half was processed for protein extraction and ELISA analysis to quantify K17 and CD31 expression, as well as for caspase-3/7 activity assays. The collected culture media were analyzed for lactate dehydrogenase (LDH) activity and measurement of IL-1α, IL-6, and IL-8 by ELISA.

Ex vivo human skin samples with standardized superficial wounds were placed on 8 µm pore membrane inserts (BD Falcon™, Durham, NC, USA) and cultured in six-well plates (BD Falcon™) at 37 °C with 5% CO_2_. Each well was filled with 2 mL of one of the following media: (i) DMEM with 10% FCS, (ii) DMEM with 10% NHS and 0.025 mg/mL of OC, (iii) CnT with 10% NHS and 0.025 mg/mL of OC, and (iv) EpiLife™ Medium (Thermo Fisher Scientific, Waltham, MA, USA) with 10% NHS and 0.025 mg/mL of OC. All media were supplemented with 100 I.U./mL penicillin and 100 µg/mL streptomycin. For each experiment, a total of 20 skin samples from the same donor were divided into four groups corresponding to the culture medium, with samples cultured for 1, 3, 6, 9, and 12 days. Culture media (1 mL) were refreshed daily. At five time points (day 1, 3, 6, 9, and 12), one milliliter of each culture medium and one skin sample from each group were collected. Part of the culture media was immediately used for LDH analysis, and the rest was frozen at −80 °C for further analyses. Skin samples were halved: one half was used for protein extraction, and the other was embedded in tissue embedding medium (HM560 Cryo-Star, Microm Labogeräte GmbH, Walldorf, Germany) for skin sectioning and histology. All samples were stored at −80 °C until further analysis. The schematic of the experimental protocol is illustrated in Figure 1.

### 2.5. Protein Extraction and Skin Sectioning Process

The protein extraction process for skin samples was conducted as described in a previous study [5]. Briefly, skin samples were cut horizontally using a microtome (Frigocut 1510 S, Leica, Bensheim, Germany) into 5 sections of 20 µm each (to collect the epidermis region) and 18 sections of 50 µm each for the dermis region. The sections were collected in a 2 mL tube, and 600 µL of extraction buffer (100 mM Tris-HCl, 150 mM NaCl, 1 mM EDTA, 1% Triton X-100) was added. The samples were vortexed for 8 s and incubated in a Thermo Mixer at 4 °C under constant shaking at 700 rpm for 90 min. Subsequently, the tissue was homogenized using a sonicator (4 °C, 37 Hz, 200 Weff) for 10 min, followed by vortexing and centrifugation at 450× *g* for 5 min. The collected supernatant was aliquoted and stored at −80 °C for subsequent caspase-3/7 activity analysis and K17 and CD31 ELISA measurements. Frozen skin samples were vertically sectioned at 7 µm thickness. The sections were collected on slides and stored at 4 °C. For each skin sample, at least eight sections containing the entire wound center and edges were prepared. These sections were subsequently used for H&E staining, immunofluorescence staining for K17 and CD31, as well as IHC staining for TGF-β2, type I collagen, type III collagen, and matrix metalloproteinase-9 (MMP-9).

### 2.6. Assessment of Skin Cell Viability and Apoptosis Using LDH and Caspase-3/7 Assays

Skin cell viability was quantified using the CyQUANT™ LDH Cytotoxicity Assay Kit (Invitrogen™, Carlsbad, CA, USA), following the manufacturer’s instructions. Culture media collected at each time point from three independent donors were used for analysis. Briefly, four supplemented culture media served as media controls. Then, 50 µL of each sample and control medium was transferred to a 96-well flat-bottom plate in triplicate, followed by the addition of 50 µL of reaction mixture to each well. The plate was incubated at room temperature for 30 min, protected from light. After incubation, 50 µL of stop solution was added to each well and gently mixed. Absorbance at 490 nm was measured using an EnSpire multimode plate reader (PerkinElmer, Akron, OH, USA), with background absorbance at 680 nm and skin-free medium control subtracted to determine sample LDH activity.

To further assess apoptosis in ex vivo skin, caspase-3/7 activity was quantified using the Caspase-3/7 Activity Fluorometric Assay Kit (UBPBio, Dallas, TX, USA) with slight modifications to the manufacturer’s protocol. Skin extracts from three independent experiments were analyzed; 25 µL of each sample lysate was mixed with 45 µL of reaction buffer and incubated at 37 °C for 10 min. The mixture was then transferred to a pre-warmed black 96-well clear-bottom plate in duplicate, with 50 µL of caspase-3/7 substrate (Z-DEVD-AFC) added to each well. Caspase-3/7 activity was measured over 2 h using a kinetic assay on a plate reader (PerkinElmer, Akron, OH, USA), monitoring the formation of the product, free AFC, by measuring the fluorescence with excitation at 400 nm and emission at 508 nm. An AFC standard curve was used to calculate the amount of AFC produced, enabling quantification of caspase-3/7 activity in each reaction.

### 2.7. Histological and Immunostaining Analysis

Hematoxylin and Eosin (H&E, Roth, Karlsruhe, Germany) staining was performed according to a standard producer protocol for histological analysis of tissue morphology. Stained tissue sections were observed and imaged using an Olympus IX 50 microscope (OLYMPUS, Hamburg, Germany).

Immunofluorescence staining was conducted to detect K17 and CD31 expression. Tissue cryosections were first blocked with a serum-free protein blocking kit (Dako, Glostrup, Denmark) for 1 h at room temperature. Fixed tissue cryosections were then incubated overnight at 4 °C with primary antibodies against K17 (1:100, Dako, Glostrup, Denmark) and CD31 (1:50, Dianova, Hamburg, Germany). After three washes with PBS, sections were incubated for 45 min at room temperature with FITC-conjugated goat anti-mouse IgG secondary antibody (1:50 in 5% FCS-PBS, Vector Laboratories, Burlingame, CA, USA). Nuclei were counterstained with propidium iodide (PI, 1.5 μM) for 20 min. Sections were washed with PBS and mounted using Vectashield medium (Vector Laboratories, Burlingame, CA, USA). Images were acquired using a confocal laser microscope (LSM 700, Carl Zeiss Ltd., Jena, Germany).

Immunohistochemistry (IHC) was performed to evaluate TGF-β2, type I and type III collagen, and MMP-9 in tissue sections. Briefly, cryosections were fixed in 4% paraformaldehyde (PFA, Sigma-Aldrich, Hamburg, Germany) for 10 min and permeabilized with 0.3% Triton X-100 (Thermo Fisher Scientific, Waltham, MA, USA). After washing with PBS, sections were blocked with normal goat serum Blocking Solution (Vector Laboratories, Burlingame, CA, USA) for 30 min. Primary antibodies were applied overnight at 4 °C as follows: TGF-β2 (1:100, Abcam Inc., Cambridge, UK), type I collagen (1:100, Proteintech, Rosemont, IL, USA), type III collagen (1:200, Abcam, Cambridge, UK), and MMP-9 (1:100, Invitrogen, Carlsbad, CA, USA). The following day, sections were washed and incubated with secondary antibodies (1:200, Vector Laboratories Inc., US) for 30 min at room temperature. Sections were incubated with VECTASTAIN ABC-AP Kit and visualized with ImmPACT Vector Red AP Substrate (Vector Laboratories, Burlingame, CA, USA), according to the manufacturer’s instructions. Slides were then counterstained with 1% Methyl Green (Sigma Aldrich, St. Louis, MO, USA) and then mounted.

### 2.8. Quantification of K17 and CD31 Using ELISA

K17 and CD31 expression levels in ex vivo skin extracts were quantified using commercially available ELISA kits (K17, Cloud-clone Corp., Katy, TX, USA; CD31, Assay Genie, Dublin, Ireland). Samples from three independent experiments were measured. For each assay, duplicate measurements were performed following the manufacturers’ instructions. Absorbance values were measured at 450 nm using an EnSpire Multimode Plate Reader (PerkinElmer, Akron, OH, USA). The obtained concentrations of K17 and CD31 were normalized to total protein content, which was determined using the Pierce 660 nm Protein Assay (Thermo Fisher Scientific Inc., Rockford, IL, USA). The values at each time point were cumulatively added and then normalized relative to the day 1 values.

### 2.9. ELISA Quantification of Cytokines in Culture Media

The concentrations of proinflammatory cytokines IL-1α, IL-6, and IL-8 in the culture media were measured using commercially available ELISA kits (IL-1α: R&D Systems, #DY200; IL-6 and IL-8: CytoSet™ CHC1263 and CHC1303, Invitrogen Corporation, Carlsbad, CA, USA), following the manufacturers’ instructions. Samples were collected from the culture medium at three time points across three independent experiments. For each cytokine assay, duplicate measurements were performed. Absorbance values were recorded using an EnSpire Multimode Plate Reader (Perkin Elmer, Akron, OH, USA). The resulting concentrations represent absolute cytokine levels in the collected media without additional normalization.

### 2.10. Statistical Analysis

In this study, ex vivo human skin models were established using skin samples from three independent donors. For the evaluation of K17, TGF-β2, and MMP-9 expression in wound sections, regions of interest (ROIs) at the wound center were analyzed from five sections per group per time point for K17 and three sections for all treatment groups across all time points for TGF-β2 and MMP-9. Additionally, for type I collagen and type III collagen, semi-quantification of intensity in the DEJ and dermis area was performed. For each section, three ROIs were selected: the wound center and two adjacent areas (left and right) near the wound center. At least nine sections per group per time point were analyzed. Data are presented as line charts or diagrams, prepared using GraphPad Prism (GraphPad Software, Version 9.5, San Diego, CA, USA). Statistical analysis was performed using SPSS 23.0 (IBM SPSS Statistics 23.0). The Kruskal–Wallis test was used for unpaired nonparametric data to evaluate differences among multiple groups, and an unpaired *t*-test was applied for pairwise comparisons of normally distributed data. Statistical significance was represented as follows: * = *p* < 0.05, ** = *p* < 0.01, and *** = *p* < 0.001. For data presented as line charts, significance between groups was denoted by different letters (a, b, c, d) or other symbols, as described in the figure legends.

## 3. Results

### 3.1. Effects of Different Culture Media Composition on Cell Migration In Vitro

**Figure 2:**
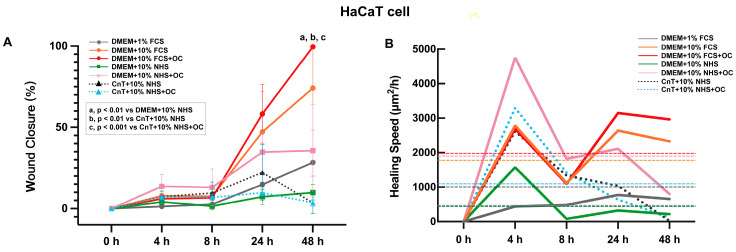
Effects of different culture medium formulations on in vitro scratch assay with HaCaT cells. (**A**) Percentage of wound closure in HaCaT cells. Wound closure quantified using ImageJ. Data are presented as mean ± SEM of areas from three independent experiments. For DMEM + 10% FCS + OC, significant differences are indicated as follows: a, compared with DMEM + 10% NHS (*p* < 0.01); b, compared with CnT + 10% NHS (*p* < 0.01); and c, compared with CnT + 10% NHS + OC (*p* < 0.001). (**B**) Wound healing speed of HaCaT cells. Data are derived from the same data set as in B and are presented as mean values; dashed lines represent the average wound healing speed in each group throughout a 48 h culture period.

**Figure 3:**
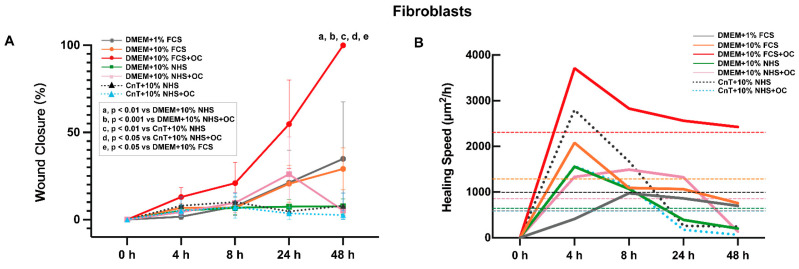
Effects of different culture media and supplements on in vitro scratch assay with fibroblasts. (**A**) Percentage of wound closure quantified using ImageJ. Data are presented as mean ± SEM from three independent experiments. For DMEM + 10% FCS + OC, significant differences are indicated as follows: a, compared with DMEM + 10% NHS (*p* < 0.01); b, compared with DMEM + 10% NHS + OC (*p* < 0.001); c, compared with CnT + 10% NHS (*p* < 0.01); d, compared with CnT + 10% NHS + OC (*p* < 0.05); and e, compared with DMEM + 10% FCS (*p* < 0.05). (**B**) Wound healing speed of fibroblasts cultured in different media over a 48 h incubation period. Data are derived from the same data set as in B and are presented as mean values of three independent experiments; dashed lines indicate the average wound healing speed in each group across the 48 h culture period.

To evaluate the impact of different culture media and supplements on cell migration, the scratch assay was conducted using HaCaT keratinocytes (Figure 2) and primary human fibroblasts (Figure 3) cultured in seven different medium formulations. DMEM with 1% FCS was compared to DMEM with 10% FCS (with or without OC) and DMEM with 10% NHS (with and without OC). In addition, the CnT medium used was supplemented with 10% NHS and with or without OCs.

For HaCaT cells, representative images of the wound closure at 0, 4, 8, 24, and 48 h for each group are shown in Figure S1 (Supplementary Materials), while the ImageJ analysis is shown in Figure 2A,B. While in the DMEM + 1% FCS group no wound closure was observed over the 48h, the DMEM + 10% FCS + OC group displayed the best healing with complete scratch closure by the end of the assay. The DMEM + 10% FCS group without OCs showed the second-best wound healing with almost complete scratch closure after 48 h. The DMEM + 10% NHS + OC showed partial wound healing, but the same medium without an OC did not exhibit notable scratch closure by the end of the assay. Finally, for the CnT + 10% NHS groups with and without OCs, the wound closure was minimal independently of the presence of OCs. The statistical analysis indicated significant differences between the DMEM + 10% FCS + OC group and the DMEM + 10% NHS group (*p* < 0.01), the CnT + 10% NHS group (*p* < 0.01), and the CnT + 10% NHS + OC group (*p* < 0.001). The fastest wound closure in the first hour was found for the DMEM + 10% NHS + OC group, while after 24 h and 48 h the fastest cell migration was measured for the DMEM + 10% FCS + OC and DMEM + 10% NHS groups (Figure 2B).

We also conducted the scratch assay using primary dermal fibroblasts and the same seven media as described for HaCaT cells. Representative images of the wound closure at 0, 4, 8, 24, and 48 h are shown in Figure S2 (Supplemental Material), while the ImageJ analysis is shown in Figure 3A,B. The DMEM + 10% FCS + OC group achieved complete scratch closure by 48 h, whereas the other groups failed to display complete closure. The next highest closure rates, although still below 50%, were observed at 24 h of incubation for DMEM + 10% FCS, DMEM + 10% NHS + OC, and the negative control group (DMEM + 1% FCS). However, after 48 h, the DMEM + 10% NHS + OC group displayed a marked decrease in closure percentage, indicating a reduction in cell viability. For the remaining groups, no significant increases in closure were observed. As shown in Figure 3B the healing speeds in all groups increased significantly during the initial 4 h, as indicated by the steep slopes of the curves, thereby suggesting rapid initial migration. However, the healing speed decreased in the next hour. Notably, the average wound closure speed was highest in the DMEM + 10% FCS + OC group, followed by the DMEM + 10% FCS group, as indicated by the dashed lines in Figure 3B.

### 3.2. Skin Morphology, Necrosis, and Apoptosis

Building upon the findings from the in vitro scratch assays, we evaluated the effects of various culture conditions on the wound healing model based on ex vivo human skin. Considering that not all donated skin tissue could be used due to tissue damage, tears, or stretch marks and that for all groups and time points we intended to use skin from the same donor, we decided to have 20 skin samples per experiment divided into four groups (i.e., culture conditions) and five time points.

**Figure 4:**
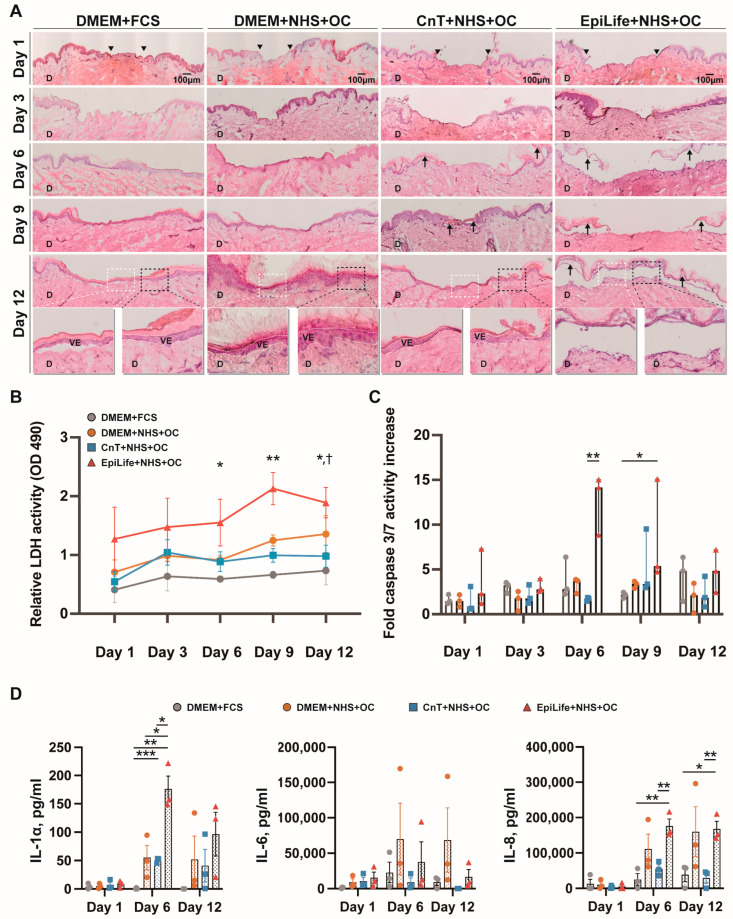
Skin morphology, necrosis, and apoptosis activities of ex vivo skin samples cultured in four different media with varying nutritional supplementation over time. (**A**) Representative images of Hematoxylin and Eosin (H&E) stained histological vertical sections showing the entire wounds at different time points. Black arrowheads indicate the wound edges immediately after the initial wounding, whereas black arrows point to epidermal–dermal detachment. The inserted images at day 12 are three-fold magnifications of the boxed area. White dotted boxes indicate the wound center at the final time point, while black dotted boxes highlight the wound edges. In the boxed images at day 12, the white double-dotted lines represent the newly formed epithelia. All pictures were captured at the same magnification (100×); scale bar, 100 µm. VE, viable epidermis; D, dermis. (**B**) Lactate dehydrogenase (LDH) release assay performed on the collected media at each time point from three independent donors. Data points represent mean ± SEM. The statistical analysis was performed using the Kruskal–Wallis test. * *p* < 0.05 and ** *p* < 0.01 indicate significant increases in the EpiLife + NHS + OC group compared with the DMEM + FCS group; † *p* < 0.05 indicates a significant increase in the EpiLife + NHS + OC group compared with the CnT + NHS + OC group. (**C**) Caspase-3/7 activity assay. Columns represent median ± SEM. Statistical differences between groups at the same time point were determined using the Kruskal–Wallis test, with significant differences indicated by * = *p* < 0.05 and ** *p* < 0.01. (**D**) Release of representative cytokines (IL-1α, IL-6, and IL-8) from ex vivo human skin in the culture media at different timepoints. Columns represent means ± SEM. Statistical significance was determined using unpaired *t*-tests. * = *p* < 0.05, ** = *p* < 0.01, and *** = *p* < 0.001.

We decided to compare the standard cell culture medium, DMEM, supplemented with 10% FCS (DMEM + FCS) with FCS-free media, DMEM, CnT, and EpiLife, supplemented with 10% NHS and 0.025 mg/mL of the OC (DMEM + NHS + OC, CnT + NHS + OC, and EpiLife + NHS + OC). We assessed the skin morphology using Hematoxylin and Eosin (H&E) staining. Representative images of skin cryosections are shown in Figure 4A. The staining revealed dynamic changes in the epidermal morphology over a 12-day culture period across the four culture conditions. The pictures of wounds at day 1 illustrate the initial wound morphology (black arrowheads mark the wound edges). The re-epithelialization in the control group (DMEM + FCS) was visible at the wound edges after 3 days of culturing, while by day 6 a thin epidermis over the entire wound surface was visible. As the culture progressed to day 9, complete re-epithelialization and new fully differentiated epidermal layers were observed. By day 12, fully re-epithelialized wounds were visible (marked in the insets by white dotted lines). The thickness of the newly formed epidermis was comparable to the adjacent epidermis at the wound edges, as shown in the insets with black dotted boxes. For the DMEM + NHS + OC group, a similar dynamic of re-epithelialization was observed, and the epidermal structure remained intact at day 12 without evident epidermal–dermal separation. For the CnT + NHS + OC group, we observed a slightly delayed re-epithelialization that was completed at day 9 but with a persisting slight epidermal–dermal detachment. In the EpiLife + NHS + OC group, full re-epithelialization was not achieved. Only at day 12 was an epidermal layer visible, but it had pronounced damages and was detached from the dermis, suggesting a significant loss of structural integrity.

To further investigate whether the differences in re-epithelialization and epidermal integrity observed across the groups were associated with variations in cell viability, the LDH activity, as a marker of membrane damage, was measured. As shown in Figure 4B, the LDH assay revealed consistently higher activity in the EpiLife + NHS + OC group compared to the other groups, beginning on day 1. A sharp increase was observed between days 6 and 9, with significant differences compared to the control DMEM + FCS group. In contrast, the other three groups exhibited relatively lower LDH activity throughout the 12-day time course. In the same samples, the caspase-3/7 activity was measured to assess the apoptosis levels, reflecting programmed cell death within the ex vivo skin tissues (Figure 4C). Between day 1 and day 12, all groups exhibited quite constant caspase-3/7 activity, with the exception of the EpiLife + NHS + OC group that showed a particularly sharp rise at day 6. The statistical analysis revealed significantly higher caspase-3/7 activity in the EpiLife + NHS + OC group compared to the CnT + NHS + OC group at day 6.

Representative cytokines, commonly released by skin cells and involved in the wound healing process, were measured in skin culture media at three time points by means of an ELISA (Figure 4D). For the DMEM + FCS group, the levels of released cytokines were low with respect to the other groups. Low concentrations of released IL-1α were measured at all time points. IL-6 peaked at day 6, while IL-8 had a small but constant increase over the 12 culture days. For the DMEM + NHS + OC group, the levels of IL-1α, IL-6, and IL-8 increased over the levels of the control group at day 6 and 12. However, a strong increase was observed only in one donor, while for the other two donors the cytokine release was more moderate, and the differences did not reach statistical significance. For the CnT + NHS + OC group, significantly higher levels of IL-1α with respect to the control were observed at day 6 and remained clearly elevated at day 12. On the contrary, IL-6 and IL-8 levels were comparable to those of the control group, DMEM+FCS. In the case of the EpiLife + NHS + OC group, both IL-6 and IL-8 levels were higher than those of the control group. Notably, a clear increase in IL-1α to levels higher than those of all other groups was measured, which confirmed the cytotoxic and disruptive events observed in the histological analyses.

### 3.3. K17 Expression and Wound Healing

**Figure 5:**
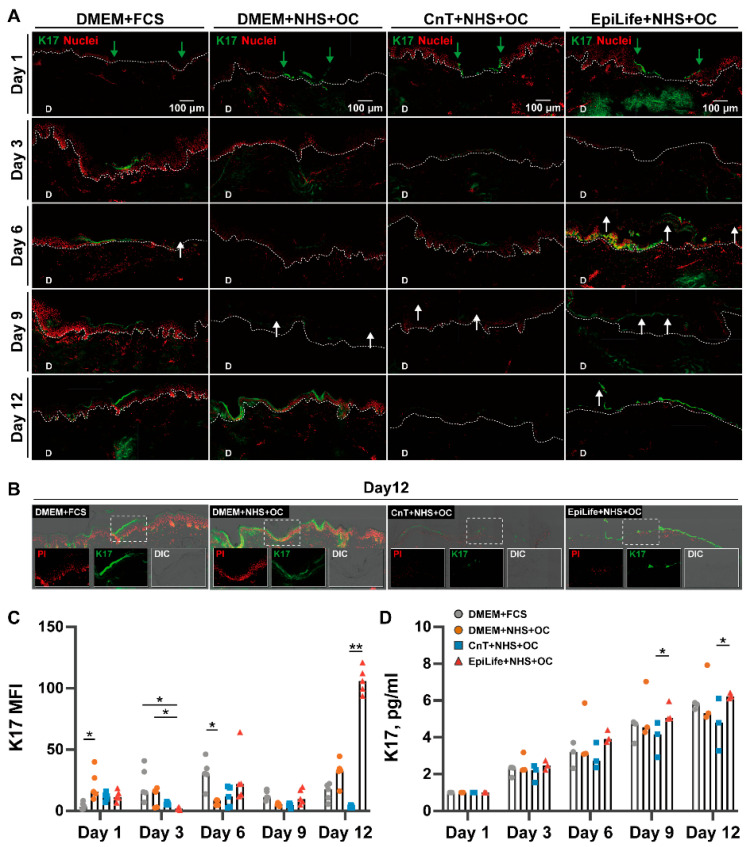
Expression of K17 in samples under different culture conditions. (**A**) Immunofluorescence staining of K17: representative images of cryosections showing cell nuclei (red) and K17 expression (green) in newly formed epidermis over a 12-day culture period. Green arrowheads indicate epithelia neogenesis, while white arrows point to epidermis when detached from dermis. White dotted lines outline the dermal–epidermal boundary. Scale bars: 100 μm (100×). (**B**) Representative fluorescence and DIC micrographs of K17-stained sections at day 12. Insert images are three-fold magnifications of the boxed area. (**C**) Mean fluorescence intensity (MFI) of K17 signal from different images from the three donors. Columns represent the median values. Statistical differences between groups at the same time point were analyzed using the Kruskal–Wallis test (* = *p* < 0.05, ** *p* < 0.01). (**D**) K17 in skin extracts collected from three donors at different time points was quantified by ELISA. Results were first normalized to the total protein content in each sample, followed by calculation of the cumulative amount of analyte versus time, further normalized to the values of each group at day 1. Data are presented as values for the three donors (symbols) and medians (columns). Statistical differences between groups at the same time point were analyzed using the Kruskal–Wallis test (* = *p* < 0.05).

Immunofluorescence staining for K17 was performed to evaluate the re-epithelialization dynamics during the wound healing process (Figure 5A). In the representative images, the green fluorescence indicates the K17 expression in the newly formed epidermis, while PI staining was employed to label the cell nuclei. In the control DMEM + FCS group, the K17 expression started at day 3, and a constant increase in the K17 signal was observed at every time point as the re-epithelialization progressed over the 12 days of incubation. In the DMEM + NHS + OC group, the keratinocyte migration into the wound started by day 1, and the formation of a weak but continuous K17 layer was visible at day 6. After 9 days of culturing, the samples exhibited well-defined re-epithelialization, characterized by a continuous K17 expression extending across the whole wound areas. After 12 days, the new epidermis was fully formed. These observations are further corroborated by the detailed differential interference contrast (DIC) micrographs that overlap with the fluorescence images (Figure 5B), which highlight the re-epithelialization in the wound center in the three-fold magnifications. The structural integrity and organization of the epidermis in the two DMEM groups are clear, reflecting superior re-epithelialization during the wound healing phase. In the CnT + NHS + OC group, the K17 expression was slightly weaker than the other groups. The EpiLife + NHS + OC group exhibited a strong K17 expression and PI staining starting from day 6. However, severe epidermal–dermal detachment (white arrows) was also observed. This disruption was accompanied by intense K17 expression in the damaged basal layers, indicating the presence of proliferating cells but with an impaired structural integrity (Figure 5A,B).

The fluorescence intensity of K17 was quantified in images from three different donors using ImageJ (Figure 5C), while the total amount of K17 in the wound was quantified in the wound tissue extracts by means of the ELISA (Figure 5D). The analyses confirmed a constant expression of K17 in the DMEM + FCS and DMEM + NHS groups, while the CnT + NHS + OC group exhibited the lowest levels during the 12-day incubation. Notably, high levels of K17 were observed in the EpiLife + NHS + OC group, which was however associated with the pronounced epidermal detachment seen in the corresponding IF and DIC images. Interestingly, the degree of the K17 expression did not reflect the integrity of the forming epidermis layers. This emphasizes the necessity to couple protein measurements like the ELISA with immunofluorescent and histological observations.

### 3.4. Angiogenesis-Related CD31 and TGF-β2 Levels

**Figure 6:**
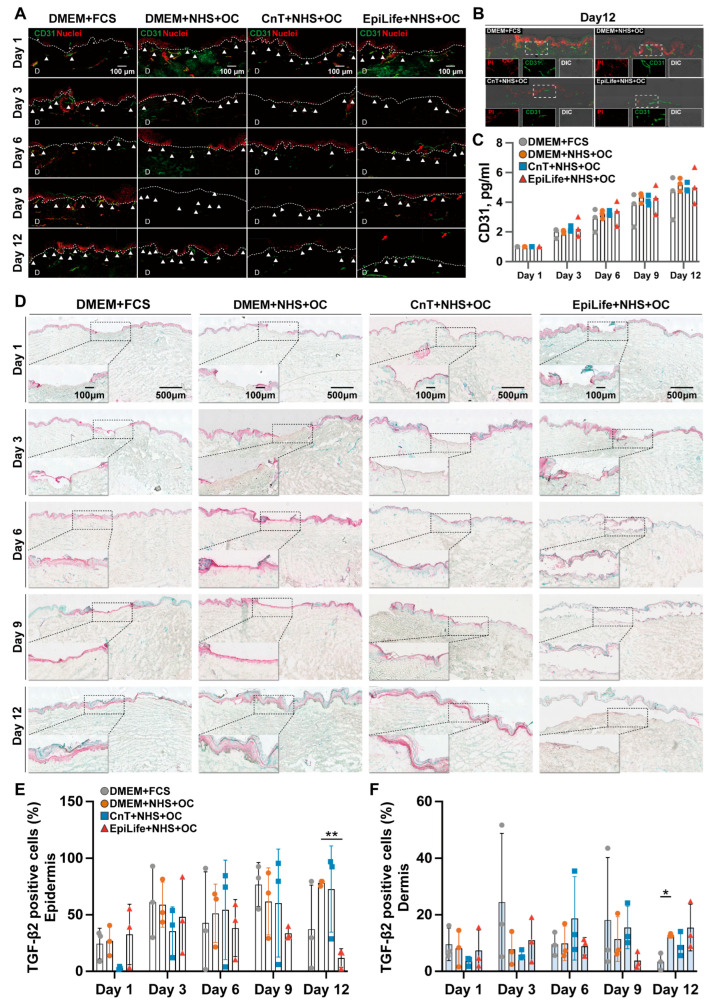
Expression of CD31 and TGF-β2 in ex vivo skin wounds over a 12-day culture period. (**A**) Immunofluorescence staining of CD31 protein. Representative images of stained frozen sections show CD31-positive endothelial cells (green), PI-stained cell nuclei (red), and overlays for each group and time point. White arrowheads indicate CD31-positive cells in the upper dermis, while red arrows point to the detached epidermis. White dotted lines demarcate the dermal–epidermal boundary. Scale bars: 100 μm. D, dermis. (**B**) Representative overlayed fluorescence/DIC micrographs of CD31 protein expressed in wound area of ex vivo skin samples on day 12. Insets are three-fold magnifications of the boxed area. Scale bars: 100 μm. (**C**) Quantification of CD31 expression in skin extracts, measured by ELISA. Data were normalized first to the total protein content in each sample and further to day 1 values. Results are expressed as the cumulative amount of analyte over time, presented as mean ± SD. (**D**) Representative images of TGF-β2 immunohistochemical staining of ex vivo skin sections at five time points. Overview images illustrate the changes in TGF-β2 expression in the epidermis and dermis across culture conditions. Black dotted boxes indicate wound centers and wound edges, with insets showing two-fold magnifications of the boxed areas. Scale bars: overview, 500 μm; inset, 100 μm. (**E**,**F**) Semi-quantification of TGF-β2-positive cells (%) in the epidermis (**E**) and dermis (**F**) across all groups and at different time points. Positive cell percentages were analyzed using QuPath software within defined ROIs from skin samples of three independent donors. Data are presented as mean ± SD (* = *p* < 0.05, ** *p* < 0.01).

Beyond examining epidermal regeneration through H&E and K17 staining, we also investigated the effect of different culture medium formulations on the equally critical process of re-vascularization. To assess this, we analyzed the expression of CD31 alongside TGF-β2. CD31 is primarily expressed on endothelial cells, including both blood and lymphatic vessels, and was observed as green fluorescence in the tissue sections, while PI (red) was used to visualize the cell nuclei (Figure 6A). CD31 expression was observed near the dermal–epidermal boundary at all time points and in all groups (Figure 6A, white arrowheads). Notably, the DMEM + FCS and DMEM + NHS + OC cultured skin samples exhibited a more prominent CD31 expression compared to the other groups, which maintained relatively lower signals. Starting from day 6, the CD31 expression became more pronounced in all groups. The EpiLife + NHS + OC group again displayed a disorganized and fragmented morphology (red arrow), with a significant detachment of the epidermal layer. By day 12, all groups demonstrated CD31 expression, with vessel-like structures clearly observed (Figure 6B). To further assess angiogenesis during wound healing, CD31 protein levels were quantified in skin extracts collected at the different time points using the ELISA (Figure 6C). Here, a slight and continuous increase in CD31 expression was observed, with comparable levels across all groups and no significant differences. Note that, whereas the immunofluorescence analysis of CD31 was focused on the superficial region of the wound, the ELISA measurement provides an evaluation of the entire upper dermis. Together, these results provide complementary insights into the CD31 expression at different time points and under different culture conditions.

Immunohistochemical (IHC) staining was performed to assess the TGF-β2 expression (Figure 6D). Representative images encompass both the wound center and the wound edge regions, providing an overview of the TGF-β2 localization. In the DMEM + NHS + OC and DMEM+FCS groups, starting from day 6, a prominent TGF-β2 expression became evident in the newly forming epidermis, particularly in the basal and suprabasal keratinocytes. In the dermis beneath the wound areas, as shown in the insert images, the TGF-β2 staining intensified around fibroblasts and vascular structures, indicating active ECM production and angiogenesis. In these groups, the TGF-β2 expression peaked at days 9 and 12, when the new epithelia were fully formed. The CnT + NHS + OC group exhibited lower TGF-β2 expression with respect to the DMEM groups in both the epidermis and dermis. On the contrary, the EpiLife + NHS + OC group showed low TGF-β2 expression at all time points along with disrupted and disorganized epidermal layers. The percentage of TGF-β2-positive cells in the epidermis and dermis of wound regions, including the normal epidermis and adjacent dermal tissue, was semi-quantitatively analyzed using QuPath software (version 0.5.1., Edinburgh University, Edinburgh, UK) across three independent donors (Figure 6E,F). In both the epidermis (Figure 6E) and the dermis (Figure 6F) of the wound area, TGF-β2-positive cells were detected, although a high variability between donors was observed. Interestingly, no clear differences were evident between the groups, except that the CnT + NHS + OC group had thinner new epithelia and that epidermal–dermal detachment occurred in the EpiLife + NHS + OC group.

### 3.5. Type I Collagen and Type III Collagen Expression

**Figure 7:**
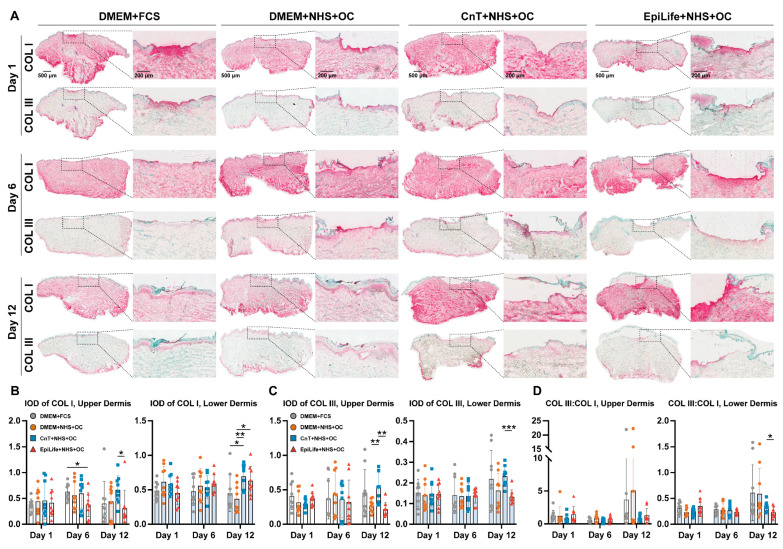
Comparative expression and semi-quantification of type I and type III collagen in ex vivo skin. (**A**) Representative images of collagen immunohistochemical staining from the same donor at day 1, day 6, and day 12. Black dotted boxes highlight the wound center, including both the epidermis and the dermis directly beneath the wound. Insert images represent a four-fold magnification of the boxed areas, scale bar = 200 μm. All overview images were taken at a total magnification of 200× with a scale bar of 500 μm. (**B**,**C**) Semi-quantification of type I (**B**) and type III (**C**) collagen intensity in the upper and lower dermis area using QuPath software. For each section, three ROIs were selected: the wound center and two adjacent areas (left and right) near the wound center. Data are presented as IOD per square micrometer (IODtreatment groups across three donors. The bar charts show type I and type III collagen IOD values for the upper and lower dermis, presented as mean ± SD. Statistical significance was determined using an unpaired *t*-test (* = *p* < 0.05 and ** = *p* < 0.01, *** *p* < 0.001). (**D**) The type III to type I collagen ratio was calculated using the IOD values obtained from semi-quantitative analysis in QuPath for the upper and lower dermis.

To further investigate the ECM deposition and remodeling processes during the wound healing of ex vivo skin cultured in different media, immunohistochemical staining for type I and type III collagen was performed on samples at days 1, 6, and 12 (Figure 7A). To ensure comparability, type I and type III collagen-stained images from the same donor were selected for each treatment group and time point. At the initial stage of wound healing (day 1), in the DMEM + FCS and DMEM + NHS + OC groups, the type I collagen staining (upper row) was more prominent and primarily distributed within the wound bed. The type III collagen staining (lower row) was also localized at the wound site, but the signal had a lower intensity. As the wound healing progressed to day 6, the type I collagen deposition in the upper wound area decreased and was similar to that in the lower dermis. Concurrently, the type III collagen expression decreased in the DMEM + FCS group, while it was still visible in the wound bed of the DMEM + NHS + OC group. By day 12, both groups exhibited a weak type I collagen expression with a higher intensity beneath the newly formed epidermis, which is indicative of stable and structured ECM remodeling. Both DMEM groups showed a uniform distribution of type III collagen-positive cells beneath the new epithelia. The CnT + NHS + OC and EpiLife + NHS + OC groups showed similar patterns but with weaker signal intensities. The only exception was for the EpiLife + NHS + OC group at day 12, where an intensive type I collagen deposition was observed. These observations correlated with a detachment of the epidermis.

To further quantify the expression of type I collagen and type III collagen in the epidermis and dermis during wound healing, integrated optical density (IOD) values were semi-quantitatively analyzed using QuPath software (Figure 7B,C). The staining intensity was measured in images from three independent donors. To ensure comprehensive coverage and minimize variability due to uneven staining, for each donor three distinct ROIs were delineated: (1) the wound center and underlying dermis, (2) the wound-adjacent epidermis and dermis on the left side, and (3) the wound-adjacent epidermis and dermis on the right side. In general, in the upper dermis, the EpiLife + NHS + OC group demonstrated lower type I collagen levels compared to the DMEM groups. On the contrary, in the lower dermis higher type I collagen levels, especially at day 6 and 12, were measured. With regard to type III collagen, no specific differences were measured between the groups with the exception of the DMEM + FCS and CnT + NHS + OC groups at day 12, which had the highest levels both in the upper and lower dermis. In addition, the type III/type I collagen ratios in the upper and lower dermis were calculated from the IOD values measured in the ROI, following evidence from previous studies suggesting that a higher ratio may contribute to scar reduction and tissue regeneration [15]. As shown in Figure 7D, the type III/type I collagen ratio in both the upper and the lower dermis exhibited minimal differences across groups during the first 9 days of the culture experiment, remaining relatively low and stable. However, at day 12, both DMEM + NHS + OC and DMEM + FCS groups demonstrated an increased type III/type I collagen ratio compared to earlier time points and to the other two groups. In contrast, lower ratios were observed in the CnT + NHS + OC and EpiLife + NHS + OC groups and were associated with irregular ECM deposition and tissue damage, as evidenced by the histological analysis, indicating delayed or compromised wound repair.

### 3.6. MMP-9 Expression

**Figure 8:**
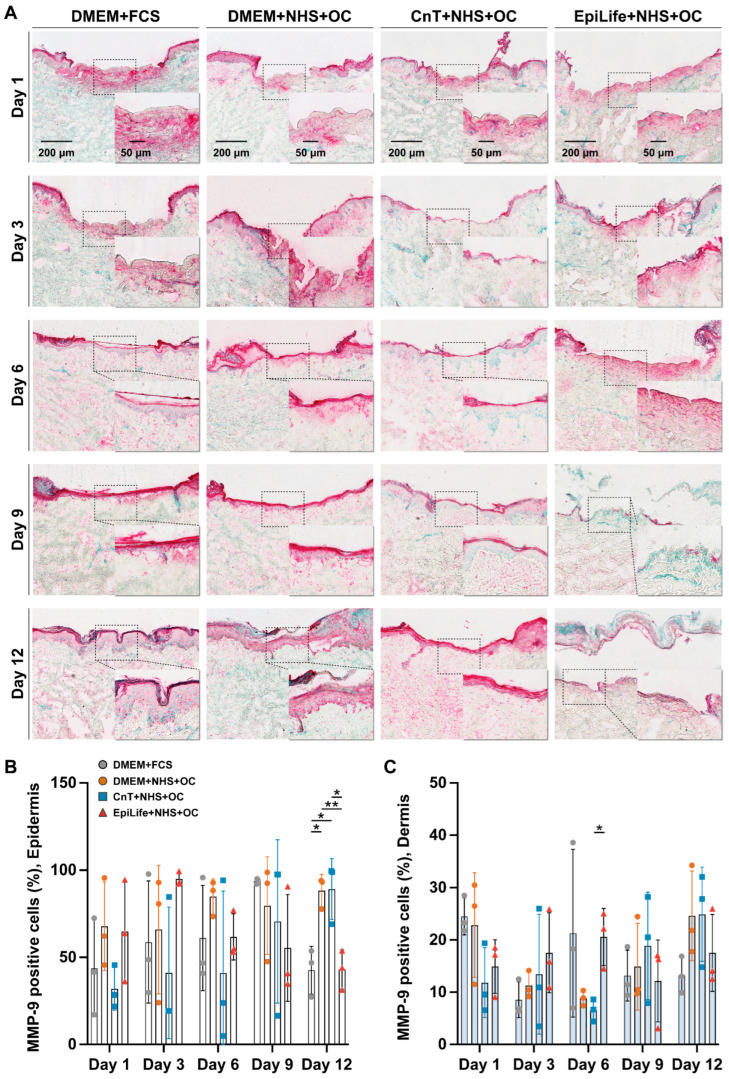
MMP-9 expression in ex vivo skin wounds during a 12-day culture period in different media. (**A**) Representative images of skin sections collected at five time points. The overview images show MMP-9 expression in the wound area and adjacent normal epidermis and dermis. Insets represent two-fold magnifications of the black dotted boxes. Scale bars: 200 μm (overview images), 50 μm (insets). (**B**,**C**) Semi-quantification of MMP-9-positive cells (%) in the epidermis (**B**) and dermis (**C**) within the wound region across all treatment groups at different time points. Positive cell percentages were analyzed using QuPath software within defined ROIs from three donors. Data are presented as symbols, and columns show mean ± SD. Statistical significance was determined using unpaired *t*-tests. * = *p* < 0.05, and ** = *p* < 0.01.

To further explore ECM remodeling during wound healing under different culture conditions, we investigated the MMP-9 expression, expanding on insights from the type I and type III collagen deposition. As a key enzyme regulating collagen degradation and the ECM turnover, MMP-9 offers valuable information on the balance between the ECM formation and remodeling, providing a deeper understanding of the influence of different culture media on ex vivo wound healing. Representative images of the immunohistochemical staining were selected from three independent donors, focusing on the wound center (Figure 8A). A two-fold magnification of the area in the inset is also shown to provide a clearer view of the localized MMP-9 expression. In the DMEM + FCS and DMEM + NHS + OC groups, during the early phase, the MMP-9 signal was localized primarily in the dermis immediately beneath the wound site, while the MMP-9 expression in deeper dermal tissues remained minimal across all groups. The MMP-9 expression in the wound decreased starting from day 6, as the re-epithelialization was already at an advanced stage. At day 12, the MMP-9 signal was low and distributed throughout the newly formed tissue. In the CnT + NHS + OC group, the MMP-9 expression was high in the wound area at day 1, while at day 3 and day 6 a decrease was observed. Starting from day 9 and peaking at day 12, the MMP-9 expression markedly increased in all of the dermis. In the EpiLife + NHS + OC group, a more pronounced MMP-9 signal in the wound bed and at the DEJ was observed at days 1, 3, and 6, whereas starting from day 9, this group exhibited weak and localized MMP-9 expression, primarily restricted to fragmented areas within the wound bed. The percentage of MMP-9-positive cells in the upper and lower wound layers was quantified in the sections using the QuPath software (Figure 8B,C). No clear trends were detectable with the exception of the increased MMP-9-positive cell percentages in the epidermis of DMEM + NHS + OC and CnT + NHS + OC groups at day 12.

## 4. Discussion

In this study, we evaluated different culture media and supplements for the culture of ex vivo human skin with the purpose of refining wound models based on skin explants. We aimed to identify animal-free alternatives to replace standard media supplemented with FCS. Since ex vivo skin samples can be used as preclinical wound models for the screening of drug delivery systems and healing molecules, our focus was mainly on skin viability and wound healing markers. DMEM supplemented with 10% FCS was selected as a reference medium as it supports cellular proliferation and migration [11,15,16] and is widely used for cell and tissue cultures. We tested two specialized media: CnT and EpiLife. The CnT medium is specifically optimized to sustain keratinocyte and fibroblast activity, ensuring reliable outcomes when culturing full-thickness 3D skin models and making it particularly suitable for co-culture systems. Its animal-free chemically defined formulation minimizes component variability, providing a robust and controlled environment for experiments that require the precise regulation of fibroblast behavior [17,18]. EpiLife, on the other hand, is a keratinocyte-specific medium designed to facilitate re-epithelialization by promoting keratinocyte migration and proliferation and can also be used in co-culture set-ups [19,20]. Normal human serum (NHS) was incorporated as an alternative to FCS. NHS provides essential growth factors, hormones, and nutrients necessary to sustain cellular activities [12]. Notably, while FCS is derived from unborn calves, NHS is derived from adult humans. Thus, NHS better mimics the biochemical and hormonal environment of human tissues by offering adult human-specific growth factors that are more compatible with the human ex vivo model. Moreover, the use of human serum minimizes potential cross-species immune reactions or inflammatory responses, which could otherwise interfere with wound healing processes [13]. Additionally, replacing animal-derived supplements with alternatives supports the 3R principles (Replacement, Reduction, and Refinement) by reducing the reliance on animal-derived products. Moreover, to further optimize the culture conditions, we incorporated an oxygen carrier protein (OC) into the medium formulations to resemble the physiological activity of hemoglobin, enhance ex vivo tissue oxygenation, and, thus, mitigate hypoxia-induced tissue degeneration, which is commonly observed in ex vivo models. Oxygen is essential for tissue survival and repair, as seen in avascular wound regions where hypoxia can impair the healing process [21]. HEMOXCell was chosen because it is plant-derived and specifically designed for cell culture applications. Unlike human hemoglobin, which binds up to four oxygen molecules, HEMOXCell can simultaneously bind 156 oxygen molecules and release them efficiently based on cellular demands, without requiring cofactors. Its non-immunogenic nature, due to the absence of cell membranes and glycosylated residues, ensures compatibility without triggering immune responses [14]. Additionally, HEMOXCell enhances oxygen diffusion while stabilizing culture media through its superoxide dismutase (SOD)-like activity, which helps mitigate oxidative stress and maintain medium quality [22].

### 4.1. FCS and OC Are Essential for In Vitro Cell Proliferation and Migration

While keratinocytes are key contributors in re-epithelialization, fibroblasts have a critical role in collagen deposition, wound contraction, and crosstalk with keratinocytes to coordinate tissue regeneration [23]. Analyzing the responses of these cell types to different medium formulations allowed us to predict possible effects on the wound healing process and select the most promising media and supplement combinations for the ex vivo study. Notably, the OC supplementation consistently provided benefits for both HaCaT keratinocytes and dermal fibroblasts compared to media without OC (Figure 2 and Figure 3). Hypoxia poses a significant challenge in vivo, ex vivo, and in vitro [24]. Consistent with these insights, the observed results suggest that OC supplementation could improve cell proliferation and better replicate the physiological oxygen environment in vivo. On the other hand, hypoxia is a condition found in diabetic foot ulcers. Thus, an OC might be omitted in wound models that should reproduce the conditions in such wounds.

The composition of the base culture media is also important for optimal cell proliferation. Despite being primarily optimized for fibroblasts, the CnT medium resulted in impaired wound closure. This was probably because of the switch from DMEM + 10% FCS (used to grow cells to confluence) to the respective experimental media immediately before the assay. Sudden changes in the nutrient composition at near confluence likely affected cell metabolism and viability, particularly in the CnT group. The type of serum further influenced proliferation. Cells cultured with NHS exhibited lower proliferation compared to those with FCS. Importantly, while FCS has been widely used to support cell growth in in vitro and ex vivo models, its strong proliferative effect might artificially promote tissue regeneration and thereby mask or moderate therapeutic effects of candidate compounds.

### 4.2. The Composition of Culture Media Influenced Ex Vivo Wound Healing, Cell Viability, and Cytokine Release

Based on the observations from the in vitro scratch assay, we proceeded to test the different culture media with the ex vivo human skin model to simulate the wound healing process in a more physiologically relevant system (Figure 1). In particular, we intended to evaluate animal-free alternatives to the conventional DMEM + 10% FCS medium. In addition to the classical DMEM-based media and CnT, we included EpiLife, a keratinocyte-specific medium. We selected both NHS and OC supplementations, as NHS alone had a poor performance in the in vitro tests. While the composition of DMEM is known, the detailed composition of CnT-Prime Airlift and EpiLife media is proprietary. However, the calcium concentration was known: while DMEM contains 1.8 mM calcium chloride, CnT has only 0.07 mM calcium chloride, and EpiLife was used without a calcium supplementation. The structural deterioration observed in the EpiLife group was accompanied by a marked increase in apoptosis [25] (Figure 4C). Concurrently, this group displayed the highest LDH levels among all groups, indicating heightened cellular membrane damage and necrosis [26] (Figure 4B). Interestingly, while LDH release primarily indicates necrosis caused by membrane damage, elevated LDH levels have also been associated with late apoptosis, also called post-apoptotic necrosis [27], indicating that the increase in LDH activity observed by day 9 could be due to such late apoptotic cells. The increase in the IL-1α release starting at day 6 correlates well with the observed epidermal–dermal detachment and epidermal cell damage. In fact, IL-1α is stored in keratinocytes and can be released immediately after skin barrier damage. In summary, these results indicate that ex vivo skin explants cultured in the EpiLife medium experienced significant cellular stress. This structural deterioration was likely due to calcium deficiency, as calcium is essential for the formation of desmosomes, and junctional complexes are critical for maintaining tissue integrity [28]. As noted by Schultz et al. [29], LDH and caspase-3/7 activity serve as reliable markers of necrotic and apoptotic cell death in response to experimental stressors, further supporting our observations under suboptimal culture conditions. Compared to the control group, the DMEM+NHS+OC and CnT + NHS + OC groups had only slightly increased LDH activity, relatively low caspase-3/7 activity, and moderate cytokine release throughout the culture period. This outcome likely reflects the combined benefits of nutrients, calcium, NHS, and OCs, which provide prolonged oxygenation and a balanced composition fostering dynamic epidermal remodeling [30]. Interestingly, although DMEM + FCS consistently supported re-epithelialization, a slight increase in caspase-3/7 activity was observed by day 12, reaching levels comparable to those in the EpiLife group. This late-stage apoptotic activity likely represents the initiation of programmed cell death within differentiating epidermal cells. Apoptosis plays a regulatory role in tissue remodeling, contributing to the removal of excess or senescent cells and refining the newly formed epidermis [25,31].

### 4.3. The Composition of Culture Media Influenced K17 Temporal Expression in the Regenerating Epidermis

K17 is a type I intermediate filament protein primarily expressed in epithelial cells, particularly in the basal layer of the epidermis. It is a stress-induced keratin, typically upregulated during injury [32,33,34,35], stress [36], or inflammation [37], and is closely associated with processes such as tissue repair and epithelial regeneration [38]. K17 is expressed exclusively in the newly forming epidermis, while the normal, uninjured epidermis has no K17 expression, making it a reliable marker for ongoing wound healing processes. The immunofluorescence and ELISA measurements (Figure 5) revealed a continuous expression of K17 over the 12 days of culturing. This observation fits well with K17’s role as part of the stress response network that helps epithelial cells adapt to injury [38].

Notably, K17 expression and re-epithelialization were particularly prominent in the DMEM + FCS group, suggesting that, as a widely used and well-established culture medium, DMEM + FCS provides optimal support for cell viability and proliferation. Its balanced composition [39,40] supports keratinocytes and their stress response mechanisms, such as K17 expression [41,42,43]. The DMEM + NHS + OC group showed similar levels of K17 compared to DMEM + FCS. NHS closely mimics the physiological environment, and studies have identified transforming growth factor-alpha (TGF-α) as a key factor in human serum, which either alone or in combination with insulin significantly enhances keratinocyte motility by activating signaling pathways such as EGFR, promoting cytoskeletal reorganization and facilitating efficient wound closure [41]. These results demonstrate that NHS can achieve effects comparable to FCS, thanks to nutrients essential for skin regeneration [44,45,46,47]. On the other hand, the elevated K17 expression observed in the EpiLife + NHS + OC group from day 6 to day 12 is likely a consequence of the severe epidermal–dermal detachment, where K17 may have been upregulated as part of the tissue’s effort to adapt to and repair the extensive structural disruption.

### 4.4. The Composition of Culture Media Influenced the Angiogenesis-Related CD31 and TGF-β2 Expression

A successful re-epithelialization process requires adequate vascularization to supply oxygen and nutrients, which are essential for sustaining keratinocyte activity and promoting tissue repair [48]. Angiogenesis, characterized by the formation of new capillaries from existing blood vessels, is tightly coordinated with epithelial restoration and involves the expression of endothelial adhesion proteins, such as CD31. The expression of CD31 not only reflects the degree of vascularization but also serves as an indicator of endothelial cell activation and blood vessel maturation [49]. Considering the immune-stained wound regions, the two DMEM groups exhibited consistent and localized CD31 signals in the upper dermis, indicating an enhanced endothelial presence. This may reflect either a more advanced stage of wound healing, with increased vessel maturation, or a stronger activation of keratinocyte migration in these groups, which is known to induce VEGF expression and promote angiogenesis [50]. In contrast, the CNT + NHS + OC and EpiLife-NHS + OC groups showed slightly reduced CD31 staining, possibly due to the delayed epithelial cell activation and insufficient VEGF induction at this stage.

While CD31 highlights the formation of neovascular structures essential for wound healing, the regulation of angiogenesis involves further factors, including the VEGF, FGF, transforming growth factor (TGF), and tumor necrosis factor (TNF) [51]. Among these, TGF-β2 is a multifunctional cytokine that regulates cell proliferation and differentiation. Its expression varies across tissues and healing stages [52]. TGF-β2 stimulation drives keratinocyte proliferation and migration during early re-epithelialization [53,54], while, in the later stages of wound healing, TGF-β2 regulates ECM remodeling by promoting fibroblast migration and differentiation into myofibroblasts and facilitating collagen deposition and ECM synthesis [55]. The TGF-β2 expression in the dermis is in general lower than in the epidermis, with excessive expression contributing to fibrosis and scar formation [56]. In the analyzed skin sections (Figure 6D), the TGF-β2 expression was mainly localized in the intact epidermis and in the newly forming epithelia, while in the dermis lower but consistent signals were detected. As re-epithelialization progressed, the DMEM + FCS and DMEM + NHS + OC groups consistently exhibited strong and sustained TGF-β2 signals in the newly forming epidermis. Especially in the DMEM + FCS group, TGF-β2 expression levels declined at the end of re-epithelialization (day 12), reflecting the transition into the tissue remodeling phase [56]. These observations correlated well with re-vascularization, well-organized epidermal layers, and the effective restoration of the epidermal barrier detected in the two DMEM groups at days 9 and 12. In comparison, the CnT + NHS + OC group exhibited a delayed but steady increase in TGF-β2 expression in the epidermis, and the EpiLife + NHS + OC group showed disorganized and less intensive TGF-β2-related staining in the epidermis. These findings show that different culture medium compositions influenced both re-vascularization and re-epithelialization, which are tightly interconnected processes.

### 4.5. The Composition of Culture Media Had an Impact on Collagen Deposition and MMP-9 Expression

Following the analysis of angiogenesis, we examined ECM deposition and tissue remodeling. ECM deposition plays a critical role in wound healing, as it provides a scaffold for cellular migration, supports tissue formation, and maintains the structural integrity of the wound site. Proper ECM remodeling ensures the timely transition from inflammation to tissue repair [57]. Among the key ECM components, type I and type III collagen are essential for the matrix formation, supporting both structural integrity and cellular function during healing [52,58]. Type I collagen, the most abundant collagen in the ECM, provides tensile strength and contributes to ECM stabilization but also to scar formation in the later stage of healing [59]. In contrast, type III collagen forms an elastic network during the initial healing phase, essential for the formation of a provisional matrix that supports cellular migration [60]. A balanced deposition of type I and type III collagen is critical for efficient tissue regeneration and wound closure [61]. The dynamic regulation of these collagen types, along with factors like MMP-9, ensures proper ECM turnover, cellular migration, and the formation of a functional epidermis and dermis [62]. Type I and type III collagen are primarily expressed by fibroblasts in the dermis, where they play essential roles in maintaining the ECM structure and facilitating tissue remodeling. In contrast, the epidermis, which consists predominantly of keratinocytes, typically produces minimal amounts of these collagens. However, during wound healing, keratinocytes contribute to ECM remodeling and locally synthesize type I and type III collagen, particularly at the DEJ and the basement membrane zone (BMZ), which facilitate epidermal cell migration and attachment during the healing process [63,64,65]. This explains the collagen signals observed in the healing epidermis of the ex vivo skin samples.

Consistent with its overall poor performance, the EpiLife + NHS + OC group exhibited reduced collagen expression, further supporting evidence of fibroblast damage or dysfunction. When observing the collagen type III/I ratios, in the deeper dermis it was below 1 (approximately 0.5) while in the upper dermis it was above 1 at all timepoints, indicating that in the wound region, the reparative and remodeling processes were ongoing during the entire incubation time. Interestingly, higher ratios were found both in the upper and lower dermis for the two DMEM-based groups at day 12. This indicates that in these two groups, the remodeling processes were still ongoing, while in the other two groups (CnT and EpiLife) the collagen turnover was less effective. Several studies suggest that a higher collagen type III/I ratio can be beneficial for scar reduction and tissue regeneration [66]. Thus, the elevated ratio in the DMEM-based groups suggests a more balanced ECM turnover and fibroblast activity. Merkel et al. reported that the fetal wound tissues with a higher collagen III content relative to collagen I exhibited a more effective healing with reduced scarring, indicating the potential benefits of an increased type III/I ratio in tissue repair [67]. Similarly, studies comparing regenerative healing process in vertebral pedicle wounds of antlers with scar healing in rats demonstrated that a higher collagen type III/I ratio is associated with faster healing and reduced scarring [68].

We also investigated MMP-9, a key regulator of ECM remodeling. MMP-9, also known as gelatinase B [69], is a zinc-dependent endopeptidase that degrades type IV collagen and other ECM components [70,71]. It facilitates keratinocyte migration and dermal remodeling [70,72], with its upregulation frequently observed in basal keratinocytes at the wound edge, where it promotes basement membrane degradation and enables efficient cellular migration across the wound bed [70,73,74]. In fact, during early re-epithelialization, keratinocytes exhibit a collagenolytic phenotype mediated by MMPs [75,76], which degrade collagen in the basement membrane [77]. As wound healing progresses to the remodeling phase, the collagenolytic activity declines, restoring the dermal–epidermal integrity and ensuring structural stability. On the other hand, in chronic wounds, elevated and prolonged MMP activity can lead to excessive ECM degradation, impairing the normal wound healing process [78,79]. Beyond to its role in ECM degradation, MMP-9 contributes to wound healing by releasing matrix-bound growth factors, including TGF-β. Notably, TGF-β2 was found to be the isoform most sensitive to MMP-9-mediated activation, and a critical mechanism for the initiation of tissue repair is indeed the activation of TGF-β by the CD44-MMP-9 complex expressed on keratinocytes [80]. This activity not only supports keratinocytes’ migration but also facilitates the release of pro-angiogenic factors, such as VEGF from ECM reservoirs, promoting vascularization. In line with these mechanisms, our data suggest that the distinct expression patterns of MMP-9 and TGF-β2 may have influenced the angiogenic and ECM remodeling outcomes in the different medium groups. Importantly, the temporal differences in the MMP-9 expression patterns may have contributed to the angiogenic disparities among the groups. In the DMEM + FCS group, early but controlled MMP-9 activity may have facilitated basement membrane degradation, keratinocyte migration, and VEGF release from the ECM, ultimately supporting re-vascularization, as evidenced by strong CD31 signals. The sustained TGF-β2 expression observed in the regenerating epidermis of both DMEM-based culture medium groups may have further enhanced epithelial activation and promoted VEGF-driven angiogenesis. In contrast, the elevated MMP-9 levels in the CnT + NHS + OC group at later stages, and the sharp early surge in the EpiLife + NHS + OC group, suggest a dysregulated remodeling process. Together, these findings illustrate that the composition of the culture media orchestrates a cascade of interdependent processes, including ECM turnover, epithelial activation, and stromal signaling, that collectively shape the angiogenic and regenerative outcome of ex vivo wound healing.

## 5. Conclusions

This study demonstrates the possibility of optimizing culture media and supplements for ex vivo human skin and wound models to enhance their physiological relevance. By utilizing three distinct basic media and varied supplementation, we observed clear differences in their ability to support the key processes of wound healing, including re-epithelialization, re-vascularization, ECM deposition, and tissue structural stability. Among the tested conditions, the use of DMEM with high concentrations of glucose and calcium, in which fetal calf serum was replaced by normal human serum and complemented with a plant-based oxygen carrier, emerged as the most balanced formulation. This setting supported keratinocyte activity, fibroblast-driven ECM remodeling, and the physiological repair of the epidermal and dermal layers. It can therefore be recommended for researchers who intend to test new wound healing candidates on human skin explants. Although this study was limited to skin from three donors, and additional donors would strengthen the conclusions, the observed trends were consistent despite the inter-donor variability, indicating biological relevance and potential generalizability. Ex vivo skin explant models do not reproduce the physiological healing of acute wounds because of inherent limitations, such as the susceptibility to infection, loss of metabolic activity, lack of vascularization, and absence of immune and hormonal signaling. However, as these are conditions found in chronic wounds due to poor blood perfusion, ex vivo wound models can be useful for the preclinical testing of treatments to improve diabetic and chronic leg ulcers. The addition of an OC was a clear benefit, mitigating hypoxia-induced stress, enhancing cellular migration, and promoting tissue repair. Nevertheless, an ex vivo skin culture without OC supplementation provides an environment that may better reflect ischemic wound regions, which are typically characterized by limited perfusion and reduced oxygen and nutrient availability. Collectively, this research established a foundation for refining the culture condition for ex vivo skin explants as chronic wound models to more accurately replicate human pathophysiological responses, providing a reliable platform for the preclinical testing of new wound healing formulations.

## Data Availability

The raw data can be shared upon request to fiorenza.rancan@charite.de.

## Supplementary Materials

The following supporting information can be downloaded at: https://www.mdpi.com/article/10.3390/pharmaceutics17121611/s1, Figure S1: Effects of different culture media formulations in the in vitro scratch assay with HaCaT cells. (A) Representative images of the wound margin at 0, 4, 8, 24, and 48 h after removing the insert. Yellow dotted lines indicate the edges of the unhealed wound; scale bar, 100 μm; Figure S2: Effects of different culture media and supplements in the in vitro scratch assay with fibroblasts. (A) Wound margins were photographed at 0, 4, 8, 24 and 48 h after culture in different media and supplements. Yellow lines show the width of unhealed wound; scale bar, 100 μm.

## Abbreviations

The following abbreviations are used in this manuscript:

DEJs	Dermal–Epidermal Junctions
FCS	Fetal Calf Serum
NHS	Normal Human Serum
OC	Oxygen Carrier
DMEM	Dulbecco’s Modified Eagle Medium High Glucose
IHC	Immunohistochemical
ECM	Extracellular Matrix
TGF-β2	Transforming Growth Factor-β2
MMP-9	Matrix Metalloproteinase-9
