# Supplementary material for: Improving the Culture of Human Skin Explants for Use in Preclinical Testing of Wound Healing Treatments
Source: Pharmaceutics. 2025 Dec 15;17(12):1611. doi: 10.3390/pharmaceutics17121611 (PMC12736455; doi:10.3390/pharmaceutics17121611)
Supplement: Supplementary file 1 [file pharmaceutics-17-01611-s001.zip › pharmaceutics-3974194-supplementary.pdf]

Supplementary material

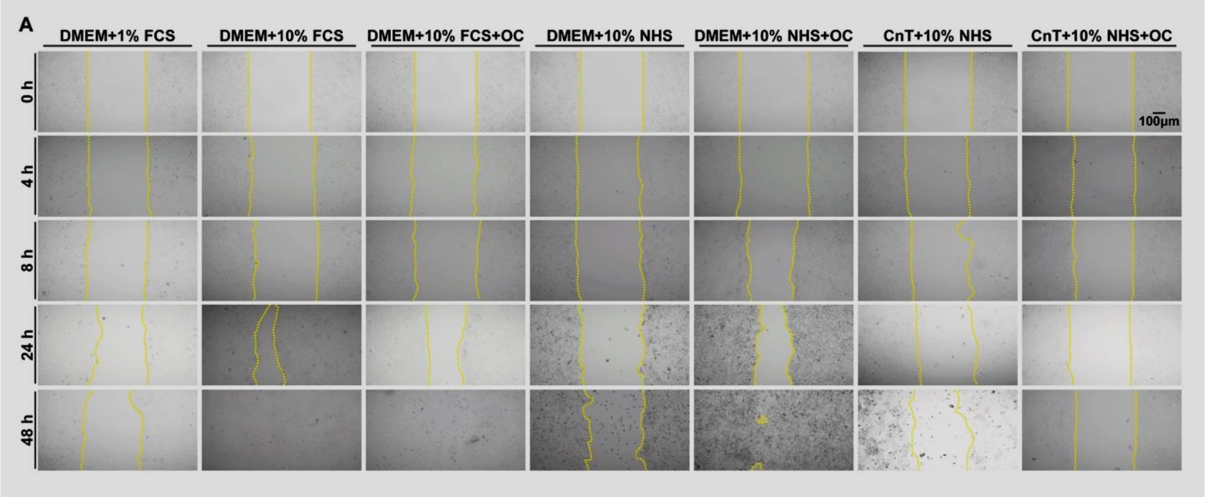

Figure S1. Effects of different culture media formulations in the *in vitro* scratch assay with HaCaT cells. (A) Representative images of the wound margin at 0, 4, 8, 24, and 48 h after removing the insert. Yellow dotted lines indicate the edges of the unhealed wound; scale bar, 100 µm.

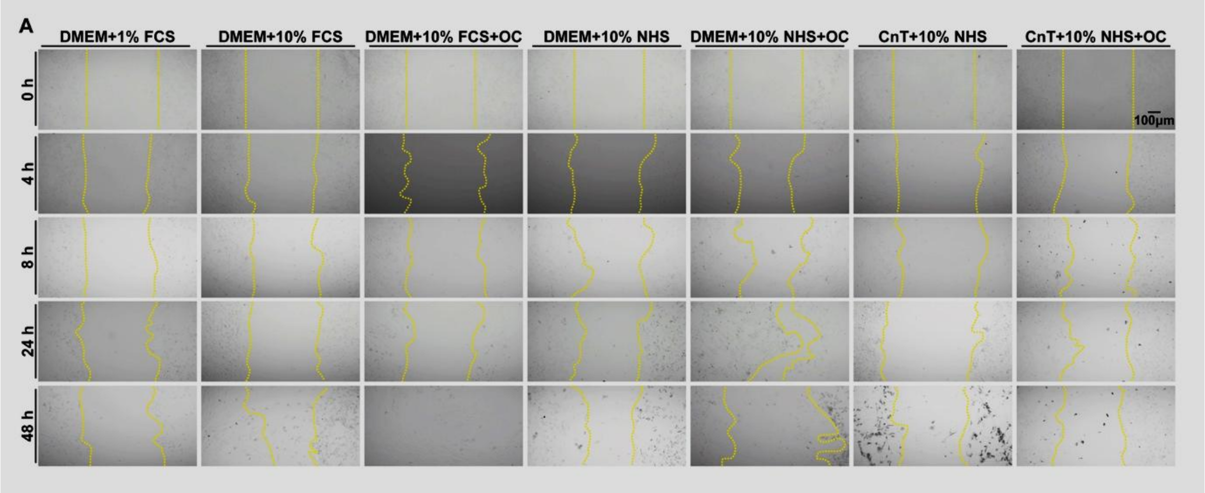

Figure S2. Effects of different culture media and supplements in the *in vitro* scratch assay with fibroblasts. (A) Wound margins were photographed at 0, 4, 8, 24 and 48 h after culture in different media and supplements. Yellow lines show the width of unhealed wound; scale bar, 100 µm.
